# Efficacy and safety of traditional Chinese medicine injections combined with FOLFOX4 regimen for gastric cancer: A protocol for systematic review and network meta-analysis

**DOI:** 10.1097/MD.0000000000027525

**Published:** 2021-10-15

**Authors:** Yanyan Zhang, Lihao Jiang, Ju Ouyang, Xianfeng Du, Longlong Jiang

**Affiliations:** a Department of Oncology, The People's Hospital of Dazu District, Chongqing 402360, China; b Department of General Surgery, The People's Hospital of Dazu District, Chongqing 402360, China.

**Keywords:** FOLFOX4 regimen, gastric cancer, network meta-analysis, traditional Chinese medicine injection

## Abstract

**Background:**

Traditional Chinese medicine injections (TCMJs) combined with FOLFOX4 regimen could achieve favorable effects in the treatment of gastric cancer. However, the efficacy and safety of different TCMJs combined with FOLFOX4 in the treatment of gastric cancer have not been fully clarified. Due to the fact that there are as many as 10 kinds of TCMJs, how to choose an appropriate TCMJ has become an urgent clinical problem. The objective of this network meta-analysis is to explore the optimal options among different TCMJs for gastric cancer.

**Methods:**

PubMed, Web of Science, Scopus, Cochrane Library, Embase, China Scientific Journal Database, China National Knowledge Infrastructure, Chinese Biomedical Literature Database, and Wanfang Data were searched to identify randomized controlled trials which focused on TCMJs combined with FOLFOX4 against gastric cancer from its inception to September 2021. Subsequently, 2 researchers will be independently responsible for literature screening, data extraction, and assessment of their quality. Standard pair-wise and Bayesian network meta-analysis will be performed to compare the efficacy and safety of different TCMJs combined with FOLFOX4 regimen via Stata 14.0 and WinBUGS1.4 software.

**Results:**

The results of this meta-analysis will be submitted to a peer-reviewed journal for publication.

**Conclusions:**

The conclusion of this systematic review will provide evidence for selecting an optimal TCMJ combined with FOLFOX4 for patients with gastric cancer.

## 1 Introduction

Gastric cancer is one of the common malignancies and is the second leading cause of cancer deaths worldwide.^[1,2]^ In the Asian region, gastric cancer is a major public health and economic burden.^[3]^ Besides, there is a poor prognosis for this disease due to late diagnosis, with an average 5-year survival rate of less than 20%.^[4]^ Surgical procedures are the preferred treatment method for gastric cancer.^[5]^ However, the early diagnosis rate of gastric cancer is low, and for patients who are inappropriate to receive surgical treatment, the multidisciplinary combination of chemotherapy-based treatment becomes the mainstay of treatment.^[6]^

The FOLFOX4 regimen is considered one of the standard chemotherapy regimens for gastric cancer.^[7,8]^ However, patients receiving chemotherapeutic drugs are subjected to treatment-related side effects, drug resistance, and adverse complications.^[9,10]^ Therefore, it is of great significance for the quality of life and prognosis of patients with gastric cancer through applying the combined treatment based on chemotherapeutic drugs to enhance the efficacy and reduce toxic side effects.^[11]^

As a complementary and alternative drug, Chinese medicine has such effects as enhancing efficacy and reducing toxicity, and it has currently become a popular drug in the treatment of gastric cancer.^[12–14]^ Chinese medicines have remarkable advantages for patients with gastric cancer in improving clinical symptoms, enhancing the efficacy of chemotherapy, reducing the toxic side effects of chemotherapy, preventing tumor metastasis and recurrence, and relieving cancer pain.^[15]^

In China, an endeavor has been made in the combination of different traditional Chinese medicine injections (TCMJs) and chemotherapy regimens.^[16,17]^ Compared with herbal tonics, TCMJs are inherently characterized by the enhanced pharmacokinetic profile and intra-tumor bioavailability.^[18,19]^ The combination of TCMJs and chemotherapy adjuvant treatment for gastric cancer could achieve a favorable effect.^[20,21]^ Specifically, the main mechanisms of TCMJs are reflected in the inhibition of tumor cell growth, induction of tumor cell apoptosis, enhancement of immunomodulatory effects, and reduction of toxic side effects caused by chemotherapeutic drugs.^[22]^

There has been effective evidence to confirm the exact efficacy of the combination of multiple TCMJs and chemotherapy in the treatment of gastric cancer.^[23]^ However, due to the wide variety of TCMJs, it is still unclear for the optimal TCMJ combined with FOLFOX4 regimen treatment strategy for patients with gastric cancer. To our knowledge, a network meta-analysis (NMA) on the comparative efficacy and safety of different TCMJs combined with FOLFOX4 regimens has not been completed previously. In an attempt to promote the rational use of TCMJs, improve safety, and provide an adequate evidence-based medical rationale, the NMA of randomized controlled trials (RCTs) reporting TCMJs combined with FOLFOX4 regimens for gastric cancer will be conducted in this study, which may help clinicians select the optimal regimen among different interventions.

## 2 Methods

### 2.1 Study registration

The protocol of this review was registered in OSF (OSF registration number: DOI 10.17605/OSF.IO/VSJ49). Besides, it was reported as per the statement guidelines of Preferred Reporting Items for Systematic Reviews and Meta-Analysis Protocol (PRISMA-P).^[24]^

### 2.2 Inclusion criteria for study selection

#### 2.2.1 Types of studies

All RCTs investigating the efficacy and safety of TCMJs combined with FOLFOX4 regimen in the treatment of gastric cancer will be included.

#### 2.2.2 Types of participants

Those patients diagnosed with gastric cancer from the pathological or cytological level will be included. There will be no restriction on gender, race, or nationality.

#### 2.2.3 Types of interventions

In the experimental group, patients with ovarian carcinoma shall be treated with TCMJs combined with FOLFOX4 regimen. While, in the control group, these patients shall receive FOLFOX4 regimen. There will be no restrictions about types of TCMJs, drug doses, frequencies, and follow-up durations.

#### 2.2.4 Types of outcome indexes

1) Primary outcomes: the clinical effectiveness rate. The clinical effectiveness rate = [the number of patients with complete responses + the number of patients with partial responses]/the total number of patients × 100%;
2) Secondary outcomes: disease-free survival and overall survival;
3) Adverse events: myelosuppression, liver and kidney function inhibition, nausea, vomiting, etc.

### 2.3 Exclusion criteria

1) Non-RCT;
2) Editorials, letters, reviews, pharmacological or chemical experiments, etc.;
3) Repeatedly detected or published literature;
4) The absence of complete data or full-text literature.

### 2.4 Data sources

All RCTs investigating the efficacy and safety of TCMJs combined with FOLFOX4 regimen in the treatment of gastric cancer published before September 2021 will be systematically searched from PubMed, Web of Science, Scopus, Cochrane Library, Embase, China Scientific Journal Database, China National Knowledge Infrastructure, Chinese Biomedical Literature Database, and Wanfang Data. The reference lists of all retrieved articles will also be manually reviewed, with the aim of identifying any relevant trails. In the light of different electronic databases, the search terms and search strategy in this study will be adjusted correspondingly, which will conduce to avoiding the problem of mismatching. The details of PubMed's search strategies are illustrated in Table 1.

**Table 1:** Search strategy in PubMed database.

Number	Search terms
#1	Stomach Neoplasms [MeSH]
#2	Cancer of Stomach[Title/Abstract]
#3	Gastric Cancer[Title/Abstract]
#4	Gastric Neoplasms[Title/Abstract]
#5	Stomach Cancer[Title/Abstract]
#6	Cancer of the Stomach[Title/Abstract]
#7	Gastric Cancer, Familial Diffuse[Title/Abstract]
#8	Neoplasms, Gastric[Title/Abstract]
#9	Neoplasms, Stomach[Title/Abstract]
#10	Cancer, Gastric[Title/Abstract]
#11	Cancer, Stomach[Title/Abstract]
#12	Cancers, Gastric[Title/Abstract]
#13	Cancers, Stomach[Title/Abstract]
#14	Gastric Cancers[Title/Abstract]
#15	Gastric Neoplasm[Title/Abstract]
#16	Neoplasm, Gastric[Title/Abstract]
#17	Neoplasm, Stomach[Title/Abstract]
#18	Stomach Cancers[Title/Abstract]
#19	Stomach Neoplasm[Title/Abstract]
#20	OR/1-19
#21	Chinese herbal injections[Title/Abstract]
#22	Traditional chinese medicine injections[Title/Abstract]
#23	Traditional chinese medicine[Title/Abstract]
#24	OR/21-23
#25	Randomized Controlled Trial[MeSH]
#26	Controlled trial[Title/Abstract]
#27	Random∗[Title/Abstract]
#28	Controlled Clinical Trial[Title/Abstract]
#29	Clinical Trial[Title/Abstract]
#30	OR/25–29
#31	#20 AND #24 AND #30

### 2.5 Data collection and analysis

#### 2.5.1 Data extraction and management

**Figure 1:**
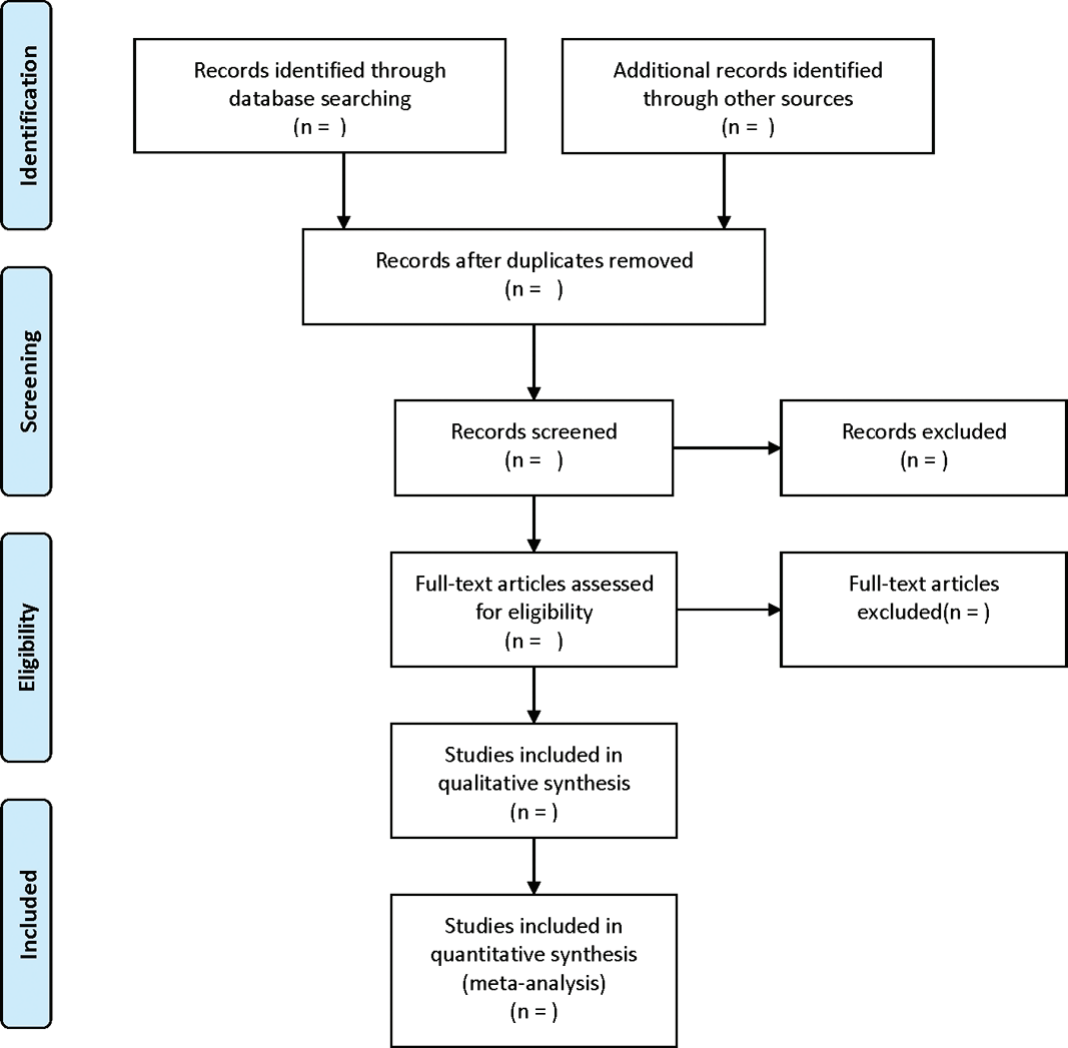
Flow diagram of study selection process.

The data will be extracted out by 2 independent reviewers in accordance with the standardized sheet recommended by the Cochrane Handbook of Systematic Reviews of Interventions. The extraction contents contain: RCT characteristics: title, name of the first author, publication date, literature sources, and quality evaluation items of RCTs. Baseline characteristics of patients: size, age, gender, tumor types, tumor stages, and so forth. Intervention: the name, dosage, and treatment cycles of TCMJs. Outcomes: the clinical effectiveness rate, disease-free survival, overall survival, myelosuppression, liver and kidney function inhibition, nausea, and vomiting, etc. If there is any inconsistent opinion, it will be further negotiated and arbitrated with a third researcher. The screening flow chart of this study is presented in Figure 1.

#### 2.5.2 Assessment of risk of bias

Two evaluators will independently evaluate the quality of the included RCTs with the Cochrane risk of bias tool (Cochrane Handbook, version 5.1.0.).^[25]^ The evaluation results will be classified into the high-risk, low-risk, and unclear categories.

#### 2.5.3 Measures of treatment effect

For dichotomous outcomes, the risk ratio will be used in the meta-analysis. All of these data will be summarized with a 95% confidence interval. The survival data will be expressed with hazard ratios and 95% confidence interval.

#### 2.5.4 Management of missing data

In case of any missing data in relevant study, the original data will be requested by email. If there is a failure in the data request, such data shall be excluded from this study.

#### 2.5.5 Assessment of heterogeneity and data synthesis

Stata 14.0 software (STATA Corporation, College Station, TX) will be used to perform the pairwise meta-analysis. Chi-square test will be performed to measure the heterogeneity among the direct comparison results, and I^2^ will be conducted to measure the heterogeneity. If there is no heterogeneity (I^2^ < 50%, *P* > .1), a fixed-effects model will be adopted in the meta-analysis; Otherwise, a random-effects model will be adopted.^[26]^ NMA will be performed with Bayesian inference (WinBUGS 1.4.3, MRC Biostatistics Unit at Cambridge, the United Kingdom). Stata 14.0 software will be used to draw relevant charts. Direct and indirect comparisons between different drug interventions will be presented by plotting a mesh relationship diagram. Bayesian inference will be performed with Markov Chain Monte Carlo to infer the posterior probabilities from the prior probabilities and to conduct estimations and inferences under the assumption that Markov Chain Monte Carlo has reached a steady state of convergence. The WinBUGS program will be run with a set number of iterations of 100,000, with the first 10,000 for annealing to remove the effect of the initial values, and a simulation chain of 3. The optimal intervention will be determined as per the probability ranking of each intervention based on the size of the surface under the cumulative ranking curve ranking probability map. Clustering analysis was performed taken into account recommending an intervention for different outcomes simultaneously to identify the optimal TCMJs.

#### 2.5.6 Assessment of reporting biases

A funnel plot will be performed to analyze the existence of publication bias if 10 or more pieces of literature are included in this meta-analysis.^[27]^

#### 2.5.7 Subgroup analysis

The subgroup analysis will be conducted during the treatment with TCMJs.

#### 2.5.8 Sensitivity analysis

The sensitivity analysis will be conducted to assess the reliability by excluding each study each time and calculating the remaining.

#### 2.5.9 Grading the quality of evidence

The Grading of Recommendations Assessment, Development and Evaluation will be adopted to evaluate the quality of evidence from the following 5 aspects: risk of bias, indirectness, inconsistency, imprecision, and publication bias.^[28]^ The quality of evidence will be graded as high, moderate, low, and very low.

#### 2.5.10 Ethics and dissemination

The contents of this paper do not involve moral approval or ethical review and will be presented in print or at relevant conferences.

## 3 Discussion

Gastric cancer is the second most common cancer with high morbidity and mortality.^[29]^ Due to the low pre-operative diagnosis rate, most patients are highly susceptible to distant metastases and about 80% of cases would eventually develop advanced gastric cancer and receive chemotherapy.^[30]^ Currently, FOLFOX4-based chemotherapy is the first-line chemotherapy regimen for gastric cancer.^[31]^ However, when chemotherapy kills and inhibits tumor cells, it also damages a considerable number of normal cells, which would induce serious complications. In recent years, the Chinese medical community has combined chemotherapeutic drugs with traditional Chinese medicine (TCM) to enhance the efficacy and reduce the toxicity of chemotherapy.^[32–34]^

TCMJs are produced by combining TCM theory with modern drug production process, and they are characterized by easy use, rapid onset of action, and high bioavailability, compared with TCM decoction.^[35,36]^ It has been demonstrated in some studies that the adjuvant use of herbal injections during chemotherapy can improve the overall efficiency and survival quality of patients with gastric cancer and reduce the incidence of adverse reactions.^[37]^

However, there is currently no NMA on the comparative efficacy and safety of different TCMJs combined with FOLFOX4 regimens. Through NMA, different interventions for treating the same disease can be quantified and analyzed, and all intervention strategies can be ranked, which contributes to determining the optimal intervention. In this study, the efficacy and safety of TCMJs combined with FOLFOX4 for gastric cancer will be summarized and ranked through NMA, which would provide a reference for determining the optimal TCMJs.

## Author contributions

**Conceptualization:** Longlong Jiang, Yanyan Zhang.

**Data curation:** Yanyan Zhang, Lihao Jiang, Ju Ouyang.

**Formal analysis:** Lihao Jiang.

**Funding acquisition:** Longlong Jiang.

**Funding support:** Longlong Jiang.

**Investigation:** Lihao Jiang.

**Methodology:** Lihao Jiang.

**Project administration:** Longlong Jiang.

**Resources:** Lihao Jiang, Ju Ouyang.

**Software operating:** Xianfeng Du.

**Software:** Ju Ouyang.

**Supervision:** Longlong Jiang.

**Validation:** Ju Ouyang, Xianfeng Du.

**Visualization:** Ju Ouyang, Xianfeng Du.

**Writing – original draft:** Yanyan Zhang and Longlong Jiang.

**Writing – review & editing:** Yanyan Zhang and Longlong Jiang.
